# Supplementary material for: The Systems Biology Research Tool: evolvable open-source software
Source: BMC Syst Biol. 2008 Jun 29;2:55. doi: 10.1186/1752-0509-2-55 (PMC2446383; doi:10.1186/1752-0509-2-55)
Supplement: Additional file 1 — SBRT Archive. An archive of the current version of the Systems Biology Research Tool. [file 1752-0509-2-55-S1.zip › sbrt-1.4.0/doc/developers_guide/api/sbrt/shell/text/chem/IrrevRxnFormatV1.html]

IrrevRxnFormatV1


|  |  |  |
| --- | --- | --- |
| **Overview** \| **Package** \| **Class** \| **Use** \| **Tree** \| **Deprecated** \| **Index** \| **Help** | |  |
| **PREV CLASS**   **NEXT CLASS** | **FRAMES**    **NO FRAMES**     **All Classes** |  |
| SUMMARY: NESTED \| FIELD \| CONSTR \| METHOD | DETAIL: FIELD \| CONSTR \| METHOD |  |


---


## sbrt.shell.text.chem Class IrrevRxnFormatV1

```
java.lang.Object
  sbrt.shell.text.chem.IrrevRxnFormatV1
```

**All Implemented Interfaces:**: Format, Formatter<IrrevRxn>, Parser<IrrevRxn>, SimpleFormat<IrrevRxn>

---

``` public final class IrrevRxnFormatV1 extends java.lang.Object implements SimpleFormat<IrrevRxn> ```

This class is used to format irreversible chemical reactions.

**Author:**
:   This class was written and documented by
    Jeremiah Wright while in the Wagner lab.

---

| **Field Summary** | |
| --- | --- |
| `static java.lang.String` | `ARROW`             The arrow used to signify an irreversible reaction. |


**Constructor Summary**

`IrrevRxnFormatV1()`
  
          Constructs a new irreversible reaction format.


| **Method Summary** | |
| --- | --- |
| `java.lang.String` | `format(IrrevRxn rxn)`             Returns a formatted string representation of the provided irreversible chemical reaction. |
| `ChemSpeciesFormatV1` | `getChemSpeciesFormat()`             Returns the format used for chemical species. |
| `LinearCombFormatV1<java.lang.String>` | `getLinearCombFormat()`             Returns the format used for stoichiometric expressions. |
| `IrrevRxn` | `parse(java.lang.String reaction)`             Parses the provided string and returns its corresponding irreversible reaction. |

**Methods inherited from class java.lang.Object**

`clone, equals, finalize, getClass, hashCode, notify, notifyAll, toString, wait, wait, wait`

**Field Detail**

### ARROW

```
public static final java.lang.String ARROW
```

:   The arrow used to signify an irreversible reaction.

    **See Also:**: Constant Field Values


**Constructor Detail**

### IrrevRxnFormatV1

```
public IrrevRxnFormatV1()
```

:   Constructs a new irreversible reaction format.


**Method Detail**

### getChemSpeciesFormat

```
public ChemSpeciesFormatV1 getChemSpeciesFormat()
```

:   Returns the format used for chemical species.

    :   **Returns:**: the format used for chemical species.

---


### getLinearCombFormat

```
public LinearCombFormatV1<java.lang.String> getLinearCombFormat()
```

:   Returns the format used for stoichiometric expressions.

    :   **Returns:**: the format used for stoichiometric expressions.

---


### format

```
public java.lang.String format(IrrevRxn rxn)
```

:   Returns a formatted string representation of the
    provided irreversible chemical reaction.

    :   **Specified by:**: `format` in interface `Formatter<IrrevRxn>`
    :   **Parameters:**: `rxn` - the reaction to be formatted. **Returns:**: a formatted string representation of the provided irreversible chemical reaction.

---


### parse

```
public IrrevRxn parse(java.lang.String reaction)
```

:   Parses the provided string and returns its
    corresponding irreversible reaction.

    :   **Specified by:**: `parse` in interface `Parser<IrrevRxn>`
    :   **Parameters:**: `reaction` - the string to be parsed. **Returns:**: the irreversible reaction corresponding to the provided string.


---


|  |  |  |
| --- | --- | --- |
| **Overview** \| **Package** \| **Class** \| **Use** \| **Tree** \| **Deprecated** \| **Index** \| **Help** | |  |
| **PREV CLASS**   **NEXT CLASS** | **FRAMES**    **NO FRAMES**     **All Classes** |  |
| SUMMARY: NESTED \| FIELD \| CONSTR \| METHOD | DETAIL: FIELD \| CONSTR \| METHOD |  |


---
